# Supplementary material for: Utilisation of Colorectal Cancer Screening Tests in European Countries by Type of Screening Offer: Results from the European Health Interview Survey
Source: Cancers (Basel). 2020 May 29;12(6):1409. doi: 10.3390/cancers12061409 (PMC7352919; doi:10.3390/cancers12061409)
Supplement: Supplementary file 1 [file cancers-12-01409-s001.pdf]

Utilisation of Colorectal Cancer Screening Tests in European Countries by Type of Screening Offer: Results from the European Health Interview Survey

Rafael Cardoso, Feng Guo, Thomas Heisser, Michael Hoffmeister and Hermann Brenner

Table 1. Odds ratio estimates and 95% CIs from random-effects subgroup meta-analyses of the association between demographic, socioeconomic, health care use and health-related factors, and faecal test use within 2 years by type of CRC screening offer.

Characteristics	(A) Nationwide organised programme with faecal tests, rollout complete		(B) Organised programme with faecal tests, partial rollout or with regional coverage only		(C) Opportunistic programme with faecal tests		(D) No programme with faecal tests or small-scale pilot only		(E) Other		p subgroup differences[d]
	OR (95% CI)	I^2 (%)	OR (95% CI)	I^2 (%)	OR (95% CI)	I^2 (%)	OR (95% CI)	I^2 (%)	OR (95% CI)	I^2 (%)	
**Demographic and socioeconomic factors**											
**Sex**											
Male	Reference		Reference		Reference		Reference		Reference		
Female	1.02 (0.94 to 1.12)	0	0.96 (0.83 to 1.12)	74	0.88 (0.76 to 1.02)	76	**0.78 (0.68 to 0.90)**	41	0.91 (0.76 to 1.08)	0	0.02
**Age (years)[a]**											
50–54	**0.68 (0.50 to 0.92)**	63	0.70 (0.49 to 1.01)	90	**0.78 (0.66 to 0.92)**	62	0.93 (0.77 to 1.11)	0			0.24
55–59	**0.84 (0.71 to 0.99)**	0	**0.71 (0.54 to 0.92)**	83	0.99 (0.90 to 1.10)	14	1.01 (0.82 to 1.25)	30			0.05
60–64	Reference		Reference		Reference		Reference		Not applicable		

65–69	**1.18 (1.05 to 1.32)**	3	1.08 (0.88 to 1.33)	80	0.93 (0.81 to 1.06)	39	**0.83 (0.70 to 0.99)**	0			< 0.01
70–74	1.16 (0.79 to 1.71)	83	**0.82 (0.68 to 0.98)**	12	0.92 (0.78 to 1.09)	56	0.84 (0.69 to 1.02)	0			0.37
**Location of residence**											
City	Reference		Reference		Reference		Reference		Reference		
Town or suburb	1.06 (0.86 to 1.29)	63	1.19 (0.97 to 1.46)	80	0.96 (0.77 to 1.18)	84	1.03 (0.87 to 1.21)	38	0.90 (0.64 to 1.25)	50	0.55
Rural area	0.89 (0.63 to 1.26)	87	1.08 (0.85 to 1.37)	86	0.98 (0.73 to 1.31)	92	0.93 (0.74 to 1.16)	65	1.02 (0.82 to 1.28)	0	0.87
**Marital status**											
Married	Reference		Reference		Reference		Reference		Reference		
Unmarried	**0.78 (0.71 to 0.86)**	0	**0.85 (0.74 to 0.97)**	63	**0.88 (0.80 to 0.98)**	50	0.99 (0.88 to 1.11)	14	0.93 (0.76 to 1.15)	8	0.04
**Education[b]**											
Lower education	0.91 (0.77 to 1.09)	58	0.86 (0.73 to 1.00)	68	1.00 (0.86 to 1.16)	69	0.99 (0.87 to 1.12)	10	1.02 (0.74 to 1.40)	30	0.57
Higher education	Reference		Reference		Reference		Reference		Reference		
**Household income[c]**											
Lower household income	Reference		Reference		Reference		Reference		Reference		
Higher household income	**1.19 (1.09 to 1.31)**	0	1.11 (0.94 to 1.30)	74	1.10 (0.99 to 1.23)	57	1.01 (0.82 to 1.24)	69	1.02 (0.77 to 1.36)	45	0.52
**Health care use**											

**Last time of a consultation with a GP**											
Less than 12 months ago	Reference		Reference		Reference		Reference		Reference		
12 months ago or longer	**0.59 (0.51 to 0.68)**	18	**0.58 (0.46 to 0.74)**	79	**0.40 (0.32 to 0.49)**	75	**0.52 (0.45 to 0.61)**	7	0.85 (0.51 to 1.41)	63	0.01
**Last time of a consultation with medical or surgical specialist**											
Less than 12 months ago	Reference		Reference		Reference		Reference		Reference		
12 months ago or longer	**0.74 (0.68 to 0.81)**	0	**0.66 (0.58 to 0.74)**	55	**0.71 (0.56 to 0.89)**	90	**0.54 (0.49 to 0.61)**	0	**0.58 (0.48 to 0.71)**	0	< 0.01
**Health-related factors**											
**Self-perceived health**											
Good or very good	Reference		Reference		Reference		Reference		Reference		
Less than good	**0.86 (0.77 to 0.96)**	19	1.05 (0.95 to 1.15)	26	1.14 (0.93 to 1.40)	86	**1.25 (1.12 to 1.40)**	1	1.10 (0.89 to 1.37)	16	< 0.01
**Lifestyle score**											
0 or 1	**0.67 (0.59 to 0.76)**	0	**0.81 (0.67 to 0.97)**	60	**0.78 (0.68 to 0.89)**	38	**0.79 (0.64 to 0.96)**	28	0.69 (0.39 to 1.21)	53	0.34
2	**0.81 (0.73 to 0.90)**	0	**0.81 (0.72 to 0.92)**	48	**0.88 (0.79 to 0.96)**	38	0.97 (0.82 to 1.15)	52	0.92 (0.73 to 1.17)	0	0.34
3 or 4	Reference		Reference		Reference		Reference		Reference		

[a] Age was not included as a covariate in the model for Finland (50–59), Ireland (50–59), Malta (50–54; 70–74), Sweden (50–59) and the UK (50–59) from category (D) and for all countries from category (E) given the narrow age ranges they comprise. [b] Lower education = upper secondary education or less; higher education = post-secondary education or more. For Malta, the categories are "lower secondary education or less" and "upper secondary education or more", respectively. [c] Lower household income = net monthly income of the household below the 2nd quintile; Higher household income = net monthly income of the household between the 2nd and 5th quintiles. [d] Test for subgroup differences (random effects). Data on at least one of the explanatory variables missing for all respondents from the Netherlands and 2881 (19%), 3477 (12%), 1444

(4%) and 3287 (9%) and 614 (13%) respondents from (**A**), (**B**), (**C**), (**D**) and (**E**), respectively. These respondents were not considered for this sub-analysis. CI, confidence interval; CRC, colorectal cancer. bold data are the statistically significant results.

Table 2. Odds ratio estimates and 95% CIs from random-effects subgroup meta-analyses of the association between demographic, socioeconomic, health care use and health-related factors, and colonoscopy use within 10 years by type of CRC screening offer.

**Characteristics**	**(A) Nationwide organised programme with faecal tests, rollout complete**		**(B) Organised programme with faecal tests, partial rollout or with regional coverage only**		**(C) Colonoscopy offered as an alternative primary screening modality**		**(D) No programme, small-scale organised programme, or opportunistic programme with faecal tests only**		***P*** **subgroup differences**[d]
	**OR (95% CI)**	***I*²(%)**	**OR (95% CI)**	***I*²(%)**	**OR (95% CI)**	***I*²(%)**	**OR (95% CI)**	***I*²(%)**	
**Demographic and socioeconomic factors**									
**Sex**									
Male	Reference		Reference		Reference		Reference		
Female	**0.87 (0.79 to 0.96)**	0	**0.85 (0.77 to 0.93)**	36	**0.81 (0.76 to 0.86)**	0	**0.90 (0.83 to 0.98)**	10	0.23
**Age (years)**[a]									
50-54	**0.62 (0.50 to 0.77)**	16	**0.63 (0.52 to 0.76)**	45	**0.50 (0.40 to 0.62)**	78	**0.73 (0.62 to 0.85)**	14	0.05
55-59	0.85 (0.71 to 1.02)	0	**0.85 (0.76 to 0.96)**	4	**0.73 (0.65 to 0.82)**	30	**0.73 (0.64 to 0.84)**	0	0.19
60-64	Reference		Reference		Reference		Reference		
65-69	1.11 (0.97 to 1.26)	0	1.11 (1.00 to 1.23)	13	1.11 (0.98 to 1.26)	38	1.10 (0.97 to 1.25)	0	1.00
70-74	1.14 (0.92 to 1.41)	46	1.12 (0.99 to 1.28)	28	1.18 (1.00 to 1.39)	60	0.99 (0.84 to 1.16)	22	0.46
**Location of residence**									
City	Reference		Reference		Reference		Reference		
Town or suburb	0.92 (0.73 to 1.15)	65	0.92 (0.79 to 1.06)	55	1.09 (0.97 to 1.23)	52	0.85 (0.73 to 1.00)	55	0.07
Rural area	0.89 (0.72 to 1.09)	56	**0.88 (0.79 to 0.98)**	32	0.99 (0.89 to 1.10)	31	0.87 (0.73 to 1.02)	63	0.33

**Marital status**									
Married	Reference		Reference		Reference		Reference		
Unmarried	**0.87 (0.78 to 0.97)**	0	0.96 (0.89 to 1.05)	16	**0.80 (0.72 to 0.89)**	52	1.02 (0.94 to 1.11)	0	< 0.01
**Education**[b]									
Lower education	0.86 (0.64 to 1.15)	82	**0.84 (0.76 to 0.93)**	22	**0.86 (0.78 to 0.94)**	25	0.87 (0.76 to 1.00)	56	0.98
Higher education	Reference		Reference		Reference		Reference		
**Household income**[c]									
Lower household income	Reference		Reference		Reference		Reference		
Higher household income	1.03 (0.92 to 1.14)	0	**1.19 (1.07 to 1.32)**	35	**1.17 (1.10 to 1.25)**	0	1.14 (1.00 to 1.30)	52	0.16
**Health care use**									
			**Last time of a consultation with a GP**						
Less than 12 months ago	Reference		Reference		Reference		Reference		
12 months ago or longer	**0.61 (0.52 to 0.72)**	0	**0.57 (0.51 to 0.64)**	0	**0.55 (0.49 to 0.61)**	34	**0.59 (0.48 to 0.72)**	60	0.73
			**Last time of a consultation with medical or surgical specialist**						
Less than 12 months ago	Reference		Reference		Reference		Reference		
12 months ago or longer	**0.59 (0.53 to 0.65)**	0	**0.59 (0.52 to 0.67)**	57	**0.56 (0.50 to 0.63)**	62	**0.47 (0.41 to 0.54)**	59	0.06

**Health-related factors**									
**Self-perceived health**									
Good or very good	Reference		Reference		Reference		Reference		
Less than good	**1.58 (1.34 to 1.86)**	54	**1.43 (1.32 to 1.53)**	0	**1.18 (1.08 to 1.29)**	29	**1.70 (1.55 to 1.87)**	10	< 0.01
**Lifestyle score**									
0 or 1	0.88 (0.76 to 1.02)	0	**0.78 (0.64 to 0.94)**	57	**0.77 (0.70 to 0.84)**	0	**0.74 (0.65 to 0.84)**	0	0.35
2	1.00 (0.89 to 1.13)	0	0.92 (0.84 to 1.01)	6	**0.86 (0.80 to 0.92)**	0	**0.80 (0.73 to 0.88)**	13	0.02
3 or 4	Reference		Reference		Reference		Reference		

[a] Age was not included as a covariate in the model for Finland (50-59), Ireland (50-59), Malta (50-54; 70-74), Sweden (50-59) and the UK (50-59) from category (D) given the narrow age ranges they comprise. [b] Lower education = upper secondary education or less; higher education = post-secondary education or more. For Malta, the categories are "lower secondary education or less" and "upper secondary education or more", respectively. [c] Lower household income = net monthly income of the household below the 2nd quintile; Higher household income = net monthly income of the household between the 2nd and 5th quintiles. [d] Test for subgroup differences (random effects). Data on at least one of the explanatory variables missing for all respondents from the Netherlands and 3003 (19%), 3992 (13%), 1605 (5%) and 3360 (9%) respondents from (A), (B), (C) and (D), respectively. These respondents were not considered for this sub-analysis. CI, confidence interval; CRC, colorectal cancer. bold data are the statistically significant results.

Table S3. Odds ratio estimates and 95% CIs from random-effects subgroup meta-analyses of the association between demographic, socioeconomic, health care use and health-related factors, and having undergone either faecal tests within 2 years or colonoscopy within 10 years by type of CRC screening offer.

**Characteristics**	**(A)) Nationwide organised programme with faecal tests, rollout complete**		**(B) Organised programme with faecal tests, partial rollout or with regional coverage only**		**(C) Colonoscopy offered as an alternative primary screening modality**		**(D) No programme, small-scale organised programme, or opportunistic programme with faecal tests only**		***P* subgroup differences[d]**
	**OR (95% CI)**	**I^2 (%)**	**OR (95% CI)**	**I^2 (%)**	**OR (95% CI)**	**I^2 (%)**	**OR (95% CI)**	**I^2 (%)**	

**Demographic and socioeconomic factors**									
**Sex**									
Male	Reference		Reference		Reference		Reference		
Female	1.01 (0.92 to 1.10)	0	0.92 (0.82 to 1.03)	62	**0.89 (0.81 to 0.97)**	47	**0.90 (0.83 to 0.99)**	36	0.18
**Age (years)[a]**									
50-54	**0.63 (0.47 to 0.85)**	60	**0.71 (0.53 to 0.96)**	86	**0.57 (0.50 to 0.64)**	34	**0.77 (0.64 to 0.92)**	52	0.04
55-59	0.86 (0.73 to 1.02)	0	**0.76 (0.61 to 0.94)**	76	**0.81 (0.74 to 0.89)**	0	0.87 (0.76 to 1.00)	26	0.68
60-64	Reference		Reference		Reference		Reference		
65-69	**1.23 (1.10 to 1.39)**	0	1.05 (0.86 to 1.27)	80	1.00 (0.86 to 1.16)	55	1.03 (0.93 to 1.16)	0	0.09
70-74	0.87 (0.55 to 1.38)	90	0.92 (0.78 to 1.09)	65	1.00 (0.78 to 1.28)	82	0.99 (0.88 to 1.11)	0	0.87
**Location of residence**									
City	Reference		Reference		Reference		Reference		
Town or suburb	0.93 (0.69 to 1.24)	82	1.02 (0.87 to 1.19)	71	1.03 (0.87 to 1.22)	76	0.90 (0.79 to 1.03)	57	0.52
Rural area	0.85 (0.58 to 1.24)	89	0.98 (0.80 to 1.19)	83	0.96 (0.77 to 1.20)	86	0.88 (0.74 to 1.05)	75	0.83
**Marital status**									
Married	Reference		Reference		Reference		Reference		
Unmarried	**0.76 (0.69 to 0.84)**	0	0.91 (0.82 to 1.01)	53	**0.76 (0.67 to 0.85)**	64	1.00 (0.93 to 1.08)	0	< 0.01
**Education[b]**									
Lower education	0.79 (0.59 to 1.05)	83	**0.82 (0.74 to 0.91)**	40	**0.91 (0.85 to 0.98)**	0	0.89 (0.79 to 1.00)	55	0.36
Higher education	Reference		Reference		Reference		Reference		

**Household income[c]**									
Lower household income	Reference		Reference		Reference		Reference		
Higher household income	**1.23 (1.06 to 1.42)**	50	1.15 (0.99 to 1.35)	78	**1.16 (1.09 to 1.24)**	0	1.10 (0.96 to 1.25)	64	0.74
**Health care use**									
**Last time of a consultation with a GP**									
Less than 12 months ago	Reference		Reference		Reference		Reference		
12 months ago or longer	**0.55 (0.48 to 0.62)**	7	**0.59 (0.52 to 0.68)**	49	**0.44 (0.37 to 0.53)**	79	**0.54 (0.46 to 0.63)**	54	0.09
**Last time of a consultation with medical or surgical specialist**									
Less than 12 months ago	Reference		Reference		Reference		Reference		
12 months ago or longer	**0.62 (0.53 to 0.73)**	62	**0.62 (0.54 to 0.71)**	74	**0.59 (0.48 to 0.71)**	88	**0.52 (0.47 to 0.58)**	49	0.16
**Health-related factors**									
**Self-perceived health**									
Good or very good	Reference		Reference		Reference		Reference		
Less than good	1.00 (0.85 to 1.18)	62	**1.22 (1.10 to 1.35)**	48	1.08 (0.97 to 1.21)	54	**1.50 (1.39 to 1.63)**	6	< 0.01
**Lifestyle score**									
0 or 1	**0.64 (0.48 to 0.86)**	76	**0.77 (0.63 to 0.95)**	72	**0.78 (0.67 to 0.92)**	61	**0.76 (0.67 to 0.85)**	9	0.70

2	**0.79 (0.66 to 0.95)**	55	**0.83 (0.75 to 0.91)**	27	**0.84 (0.78 to 0.91)**	17	**0.87 (0.79 to 0.95)**	32	0.83
3 or 4	Reference		Reference		Reference		Reference		

[a] Age was not included as a covariate in the model for Finland (50-59), Ireland (50-59), Malta (50-54; 70-74), Sweden (50-59) and the UK (50-59) from category (D) given the narrow age ranges they comprise. [b] Lower education = upper secondary education or less; higher education = post-secondary education or more. For Malta, the categories are "lower secondary education or less" and "upper secondary education or more", respectively. [c] Lower household income = net monthly income of the household below the 2nd quintile; Higher household income = net monthly income of the household between the 2nd and 5th quintiles. [d] Test for subgroup differences (random effects). Data on at least one of the explanatory variables missing for all respondents from the Netherlands and 2895 (19%), 3839 (13%), 1562 (5%) and 3186 (9%) respondents from (A), (B), (C) and (D), respectively. These respondents were not considered for this sub-analysis. CI, confidence interval; CRC, colorectal cancer. bold data are the statistically significant results.

Table 4. Healthy lifestyle score.

Lifestyle score	Points	Description
Smoking[a]	0	Daily smoking
	1	Nonsmoking or occasional smoking
Alcohol[b]	0	≥2 drinks per day
	1	<2 drinks per day
Physical activity[c]	0	<150 minutes per week
	1	≥150 minutes per week
BMI	0	Overweight or obese (BMI ≥25 kg/m^2)
	1	Normal weight (18.5 kg/m^2 ≤ BMI <25 kg/m^2)

[a] Tobacco products (manufactured cigarettes, hand-rolled cigarettes, cigars, pipes, etc.). [b] Consumption of alcoholic drinks of any kind (e.g. beer, wine, cider, spirits, cocktails, premixes, liqueurs, homemade alcohol) on average on one of the days (Monday to Thursday). [c] It includes time spent on doing sports, fitness or recreational physical activities in a typical week. BMI, body mass index. Respondents with BMI <18.5 kg/m2 were not considered for the lifestyle score.

Table 5. Categorisation of countries/ age groups by type of colorectal cancer screening offer [a].

**Analyses of faecal test use**		
**Type of CRC screening offer**	**Country**	**Age group (years)**
(A) Nationwide fully rolled out organised programmes with faecal tests	Croatia	50–74
	France	50–74
	Slovenia	50–69
	UK	60–74
(B) Organised programmes with faecal tests in partial rollout or with regional coverage only	Belgium	50–74
	Czech Republic	50–74
	Denmark	50–74
	Finland	60–69
	Ireland	60–69
	Italy	50–69
	Lithuania	50–74
	Malta [d]	55–64
	The Netherlands	55–74
	Spain	50–69
	Sweden	60–69
(C) Opportunistic programmes with faecal tests [b]	Austria	50–74
	Germany	50–74
	Greece	50–74
	Latvia	50–74
	Luxembourg	50–74
	Portugal	50–74
	Slovakia	50–74
(D) No programme with faecal tests or small-scale pilot programme only	Bulgaria	50–74
	Cyprus	50–74
	Estonia	50–74
	Finland	50–59

Analyses of colonoscopy use and use of either faecal tests or colonoscopy		
Type of CRC screening offer	Country	Age group (years)
(A) Nationwide fully rolled out organised programmes with faecal tests	Croatia	50–74
	France	50–74
	Slovenia	50–74
	UK	60–74
(B) Organised programmes with faecal tests in partial rollout or with regional coverage only	Belgium	50–74
	Denmark	50–74
	Finland	60–74
	Ireland	60–74
	Italy	50–74
	Lithuania	50–74
	Malta	55–69
	The Netherlands	55–74
	Spain	50–74
	Sweden	60–74
(C) Colonoscopy offered as an alternative primary screening modality	Austria	50–74
	Czech Republic	50–74
	Germany	50–74
	Greece	50–74
	Iceland [e]	50–74
	Luxembourg	50–74
	Portugal	50–74
	Slovakia	50–74
(D) No programme, small-scale organised programme, or opportunistic programme with	Bulgaria	50–74
	Cyprus	50–74
	Estonia	50–74
	Finland	50–59

	Hungary	50–74	faecal tests as the first-line method only	Hungary	50–74
	Iceland	50–74		Ireland	50–59
	Ireland	50–59		Latvia	50–74
	Malta	50–54; 70–74		Malta	50–54; 70–74
	The Netherlands	50–54		The Netherlands	50–54
	Norway	50–74		Norway	50–74
	Poland	50–74		Poland	50–74
	Romania	50–74		Romania	50–74
	Sweden	50–59		Sweden	50–59
	UK [c]	50–59		UK [c]	50-59
(E) Other, i.e. no programme among the indicated age groups, but available in the preceding age groups	Finland	70–74			
	Ireland	70–74			
	Italy	70–74			
	Malta	65–69			
	Slovenia	70–74			
	Spain	70–74			
	Sweden	70–74			

[a] This categorisation represents the status of colorectal cancer screening implementation at the time the EHIS was carried out. [b] This group also includes countries where small-scale, pilot, organised programmes are in place, but where CRC screening is offered mainly in an opportunistic manner. [c] In Scotland, faecal tests are also offered to the 50-59-year age group. [d] In Malta, the screening programme targets the age group 55-66 years. [e] In Iceland, colonoscopy was the only screening modality offered.
